# Tanshinone IIA alleviates liver fibrosis by suppressing hepatic stellate cell proliferation via ERK/cyclin D1/p-Smad3L signaling axis

**DOI:** 10.22038/ijbms.2025.83092.17962

**Published:** 2025

**Authors:** Wenjing Liao, Fang Wu, Zhiyuan Hao, Rui Ye, Chenfei Liu, Jinglei Wu, Min Wu, Xiaoman Zhou, Mingze Sun, Yuwei Liu, Meng Fang

**Affiliations:** 1 School of Medicine, Jianghan University, Wuhan 430056, China; 2 School of Medicine, Jingchu University of Technology, Jingmen 448000, China

**Keywords:** ERK/cyclin D1/p-Smad3L – signaling, Hepatic stellate cells, Liver fibrosis, Tanshinone IIA, TGF-β1

## Abstract

**Objective(s):**

Liver fibrosis (LF) is a critical stage in chronic liver disease progression, and effective therapeutic drugs are currently lacking. Tanshinone IIA (Tan IIA), a monomer extracted from *Salvia miltiorrhiza*, shows potential in treating LF. This research aims to discuss the antifibrotic efficacy and underlying pharmacological mechanism of Tan IIA.

**Materials and Methods:**

The *in vivo* model was induced with CCl_4_ to form a LF model in mice, and the *in vitro* model was induced by TGF-β_1_ in LX-2 and HSC-T6 cells. Liver pathology was characterized by HE, Masson, and Sirius red staining, and serum levels of ALT, AST, LDH, and γ-GT were examined. Cell viability and proliferation were detected by Cell Counting Kit-8 and colony formation assays. Cell cycle distribution was detected by flow cytometry. The protein levels of p-ERK, cyclin D1, CDK4, and p-Smad3L were assessed through Western blot, immunohistochemistry, or immunofluorescence assays.

**Results:**

Tan IIA markedly decreased serum levels of ALT, AST, LDH, and γ-GT. Collagen I and α-SMA were reduced, as shown by *in vitro* and i*n vivo* models. Moreover, while arresting HSCs in the G1 phase was increased, Tan II A markedly inhibited cell viability and colony formation. Mechanistically, Tan IIA decreased the expression of p-ERK, cyclin D1, CDK4, and p-Smad3L proteins in TGF-β_1_-activated cells and CCl_4_-induced mice.

**Conclusion:**

Tan IIA may improve LF by regulating the signaling axis of ERK/cyclin D1/p-Smad3L, thereby blocking activated HSCs in the G1 phase and inhibiting their proliferation.

## Introduction

Liver fibrosis (LF) represents a self-repair and injury-healing response induced by acute or chronic liver damage (1). One of its main characteristics is extracellular matrix (ECM) deposition. The occurrence, progression, and regression of LF are largely dependent on hepatic stellate cells (HSCs), which secrete profibrotic factors (2). The activation of HSCs can result in excessive production of alpha-smooth muscle actin (α-SMA) and collagen type I alpha 1 (col1α1), promoting ECM deposition (3). Therefore, preventing HSCs from proliferation and activation has become a primary strategy in antifibrotic therapies (4, 5).

The most effective profibrotic factors are transforming growth factor-beta 1 (TGF-β_1_), which is tightly linked to the activation of HSCs. On the one hand, it can transmit signals through the classical TGF-β_1_/Smad signaling pathway, increasing collagen deposition and promoting LF progression (6). On the other hand, it may accelerate HSCs proliferation and activation through the activation of non-classical Smad pathways, including the extracellular signal-regulated kinase (ERK) pathway, thereby exacerbating the occurrence of LF by promoting ECM deposition (7). Some studies have identified cyclin D1 as a crucial target for regulating the ERK signaling pathway (8). By specifically binding to the cyclin-dependent kinase CDK4 to form a cyclin D1-CDK4 complex, cyclin D1 can further phosphorylate the linker region of Smad3 (9). This interaction creates crosstalk between the two signaling pathways, promoting mitosis and accelerating cell proliferation (10). We hypothesized that the proliferation of activated HSCs adjusts the ERK/cyclin D1/p-Smad3L signaling axis, which may be a new way to prevent and treat LF.

LF exhibits a degree of reversibility, making the identification of effective drugs crucial before its progression to cirrhosis or liver cancer (11). Tanshinone IIA (Tan IIA) is a lipophilic monomer extracted from *Salvia miltiorrhiza*, a traditional Chinese medicine. Tan IIA has been recognized for its various anti-inflammatory, antioxidant, and anti-angiogenic effects (12, 13). Additionally, Tan IIA shows promise in the treatment of LF. Some researchers found that Tan IIA can inhibit TGF-β_1_-induced HSCs proliferation and activation *in vitro *(14, 15). *In vivo* research has demonstrated that Tan IIA can inhibit the deposition of ECM and collagen, alleviating CCl_4_-induced liver injury and fibrosis in mice (16-19). However, the exact mechanism by which Tan IIA affects LF remains to be fully elucidated.

The research aims to investigate whether Tan IIA can inhibit the proliferation of activated HSCs, thereby providing an antifibrotic effect through the ERK/cyclin D1/p-Smad3L signaling axis in CCl_4_-induced mouse models and TGF-β_1_-induced LX-2 and HSC-T6 cell models.

## Materials and Methods

### Chemicals and antibodies

Tan IIA (purity: 98.0%) was acquired from Push Biotechnology Co. (Chengdu, China). Dulbecco’s Modified Eagle’s Medium (DMEM) was sourced from Gibco (Grand Island, NY, USA), and Fetal Bovine Serum (FBS) was obtained from Zhejiang Tianhang Biotechnology Co., Ltd. (Huzhou, China). Antibodies for GAPDH, cyclin D1, CDK4, collagen I, ERK, and p-ERK were supplied by Cell Signaling Technology, Inc. In contrast, the anti-p-Smad3L antibody was obtained from Immuno-Biological Laboratories Co., Ltd., and the anti-alpha smooth muscle actin antibody was acquired from Beijing Bioss Biotechnology Co., Ltd.

### Animals models

48 C57BL/6J mice were supplied by SPF (Beijing) Biotechnology Co., Ltd., weighing 18–20 g and aged 6–8 weeks (certificate of quality was No. SCXK (Beijing) 2019-0010). The mice were placed in ventilated plastic cages with a 12 hr light cycle and under controlled conditions around 22 °C with 40–60% relative humidity. All mice had free access to food and water. The study was approved by the Experimental Animal Ethics Committee of Jianghan University (Approval code: No. JHDXLL2022-036). Mice were randomized into six distinct groups **(16)**: Control, Model, CCl₄ + 10 mg·kg⁻¹ Tan IIA (Tan IIA 10), CCl₄ + 20 mg·kg⁻¹ Tan IIA (Tan IIA 20), CCl₄ + 40 mg·kg⁻¹ Tan IIA (Tan IIA 40), and CCl₄ + 0.2 mg·kg⁻¹ colchicine (Colchicine). The Control and Model groups received an equivalent volume of CMC-Na daily. All treatments continued for 12 weeks (IG). During this time, the Control group received olive oil twice a weekly. In contrast, the remaining five groups were treated with 20% CCl₄ (diluted in olive oil) twice a weekly, administered (IG) over the same 12-week period. Following the final treatment, mice were fasted overnight. After being anesthetized with isoflurane, they were weighed and subsequently euthanized through cervical dislocation. 

### Cell culture

LX-2 and HSC-T6 cells (Wuhan Pricella Biotechnology Co., Ltd.) were cultured at 37 °C with 5% CO_2_ and using DMEM supplemented with 10% FBS.

### Histological analysis

The left lobe of liver was preserved in a 4% paraformaldehyde solution and embedded in paraffin. Sections of 5 μm thickness were prepared for Masson, HE, and Sirius Red experiments. Then, histomorphological alterations were scrutinized under a microscope.

### Cell Counting Kit8 (CCK-8) assay

To perform a series of treatments on the cells randomized into six distinct groups **(**20, 21**):** Control (no Tan IIA or TGF-β_1_); TGF-β_1_ (1.5 ng/ml TGF-β_1_); Tan IIA 10 (1.5 ng/ml TGF-β_1_+10 µM Tan IIA); Tan IIA 20 (1.5 ng/ml TGF-β_1_+20 µM Tan IIA); Tan IIA 40 (1.5 ng/ml TGF-β_1_+40 µM Tan IIA); and Colchicine (1.5 ng/ml TGF-β_1_+0.5 µM Colchicine). The cells were treated for either 24 or 48 hr. Then, each well received 10 µl of CCK-8 (Beyotime, Shanghai, China) and incubated for two hours. Finally, the absorbance at 450 nm was measured using a microplate reader.

### Colony formation assay

In a 6-well plate, the cells were plated at a concentration of 1 × 10^3^ cells per well after being digested. Following cell adhesion, the cells were treated with drugs for 24 hr. 10% FBS regular culture media was used instead of the original medium and changed every 2–3 days. Crystal violet staining was performed when most colonies comprised a cluster of 50 or more cells. Following PBS wash, cells were fixed for 20 min in 4% paraformaldehyde. After PBS washing, 500 μl of crystal violet staining solution per well was added. After a 10-minute room temperature reaction, it was washed with PBS until no residual crystal violet color remained. Photos were taken with a scanner.

### Cell cycle assay based on flow cytometry

After being digested, the well-growing cells were cultured in a six-well plate. Drugs were added after the cells adhered to the bottom. Then, the cell culture supernatant was transferred to pre-numbered 15 ml centrifuge tubes. After washing with PBS, 500 μl of trypsin without EDTA was added to each well to digest cells. After the cells were resuspended, the supernatant was discarded after centrifuging for 5 min at 3000 rpm. To mitigate the impact of trypsin-containing trypsin red on subsequent staining results, PBS washes were employed, with careful attention to maintaining slow and gentle movements in order to avoid excessive cell fragmentation. Post-washing, 1 ml of PBS and 3 ml of pre-chilled anhydrous ethanol were added to fix the cells, along with 3% fetal bovine serum to protect the cells. 

The fixed cells also needed to be centrifuged (1000 rpm, 5 min) after a 24 hr fixation at 4 °C, and the supernatant was discarded. After adding 500 μl of the prepared propidium iodide staining solution, the cells were incubated in the dark for 30 min. After the cells were filtered through a 300-mesh nylon mesh, we used a flow cytometer to analyze them, followed by data analysis using Modifit 5.0 software.

### Immunohistochemistry assay

After dewaxing and hydration, the paraffin sections were placed in a sodium citrate buffer solution for antigen retrieval. These sections were sealed using a 5% FBS solution. The samples were cultured with different primary antibodies (CDK4 1:50; p-Smad3L 1:25; collagen I 1:50) at 4 °C overnight. After PBS washing, the samples were treated with secondary antibodies for half an hour. Stained with DAB solution, cell nuclei were counterstained with hematoxylin, blued with running tap water, and finally sealed with neutral resin. 

### Immunofluorescence staining

The cells were cultured in cell slides. After drug treatment, they were fixed in 4% paraformaldehyde for 10 min. The cell slides were permeabilized using 0.5% Triton X-100 for 20 min. Blocking was performed with 5% FBS and incubated with p-Smad3L (1:250) or CDK4 antibody (1:250) overnight at 4 °C. Following washed by TPBS, the samples were cultured with secondary antibodies: Alexa Fluor 488-labeled goat anti-rabbit IgG or 555-labeled donkey anti-rabbit IgG, both at a dilution of 1:100 (Beyotime, Shanghai, China). After another round of washing, Hoechst (Beyotime, Shanghai, China) was used to stain for 20 min. At last, fluorescence images were taken using an inverted fluorescence microscope.

### Western blotting assay

The cells and liver tissues were lysed and extracted using an appropriate volume of lysis buffer, followed by protein quantification measured via a BCA assay. Proteins were transferred to a PVDF membrane (Millipore; Merck KGaA) after being separated by SDS-PAGE. We used 5% fat-free milk powder to block, then incubated the membrane with specific antibodies at 4 ℃ overnight: GAPDH (1:1000), cyclin D1 (1:1000), CDK4 (1:1000), α-SMA (1:1000), collagen I (1:500), ERK (1:1000), p-ERK (1:1000) and p-Smad3L (1:250). Subsequent incubation with secondary antibodies (mouse or rabbit) was performed for one hour. After the protein bands were identified using the chemiluminescence detection kit, they were subsequently observed through a chemiluminescence imaging apparatus (Bio-Rad, USA). Images J was used to analyze the images.

### Statistical analyses

The experiments were performed in triplicate, and the results are presented as mean ± standard deviation. Data analysis was performed using one-way analysis of variance (ANOVA) followed by Tukey’s post hoc test, utilizing GraphPad Prism 9. Statistical significance was set at P<0.05, with P<0.01 indicating a more pronounced difference. 

## Results

### Effects of Tan IIA on liver function indices

In the present study, the relative liver index in the model group was clearly higher than in the control group (*P*<0.01). This phenomenon suggests that after CCl_4_ treatment, the livers of the mice were damaged and hypertrophied to some extent, reflecting impaired liver function. Following Tan IIA treatment, the relative liver index of the mice in the model group decreased, with more pronounced decreases at dosages of 20 mg and 40 mg (*P*<0.01). Furthermore, compared to the control group, administration of CCl_4_ caused significantly higher serum levels of ALT, AST, γ-GT, and LDH (*P*<0.01). In contrast, Tan IIA treatment, particularly at a dosage of 40 mg (*P*<0.01), led to reduced levels of these enzymes in serum, demonstrating a significant attenuation of enzyme activity relative to the CCl_4_ group (Table 1). 

### Effects of Tan IIA on liver morphology and histopathology in CCl4-induced C57BL/6J mice

**Figure 1:**
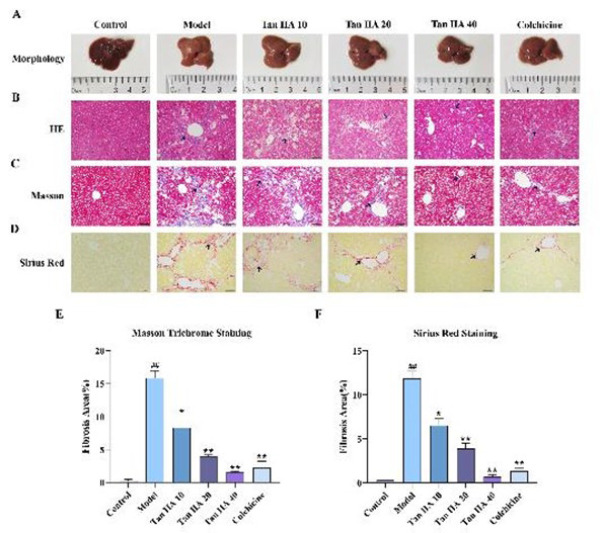
Effects of Tan IIA on liver morphology and histopathology in CCl_4_-induced C57BL/6J mice

Macroscopic examination of mouse livers revealed distinct differences among the groups: the control livers exhibited a dark red, smooth surface with a soft texture, while the livers from the model group displayed a rougher surface and a slightly harder texture. However, in mice treated with varying doses of Tan IIA, the degree of liver surface roughness gradually decreased, and the texture became softer (Figure 1 A). Histological analysis through HE staining further elucidated these observations. The mouse hepatocyte in the control group were arranged neatly, uniformly, and with an arrangement around the central vein. In contrast, livers from the model group exhibited disordered liver cells, substantial inflammatory cell infiltration at the peripheries of liver lobules and portal areas, and increased proliferation of fibrous connective tissue. Compared to the model group, the deposition of collagen fibers and infiltration of inflammatory cells decreased with increasing drug dosage, with a notable effect also observed in the positive drug group (Figure 1 B). Collagen deposition in liver tissue was assessed by Masson staining and Sirius red staining. Collagen deposition was visibly more in the livers of the model group compared to the control group (*P*<0.01), whereas Tan IIA treatment diminished collagen accumulation in a dose-dependent way (*P*<0.05 or *P*<0.01, Figure 1C-F). Reduction of LF under Tan IIA intervention showed a dose-dependent effect. 

### Effects of Tan IIA on the expression of α-SMA and collagen proteins

**Figure 2:**
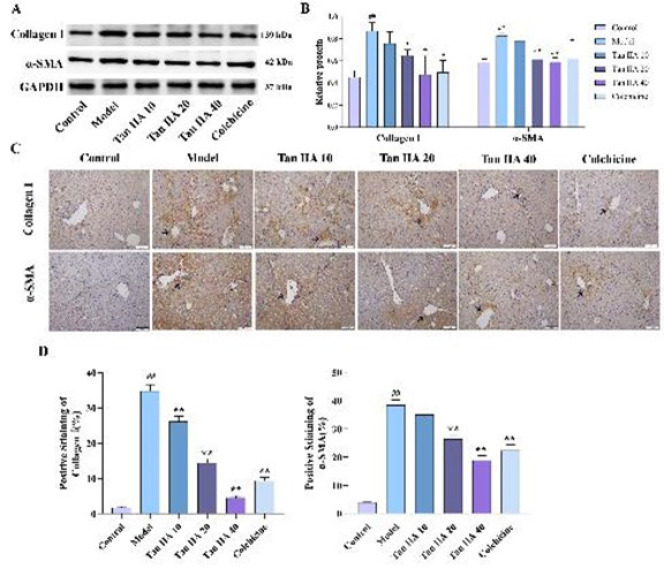
Effects of Tan IIA on α-SMA and collagen I expressions in liver tissue of mice

The progressive LF and increased liver injury are directly related to the expression of markers of activated HSCs. In this research, we assessed α-SMA and collagen I expression, the key indicators of HSC activation by western blotting and immunohistochemistry experiments. Compared to the control group, CCl_4_ treatment markedly increased α-SMA and collagen I levels (*P*<0.01). However, the expression of these proteins was concentration-dependently suppressed by Tan IIA (*P*<0.01), and a similar result was found in the colchicine group (*P*<0.01) (Figure 2 A-D). 

### Effects of Tan IIA on the expression of ERK/cyclin D1/p-Smad3L axis-related proteins in vivo

**Figure 3:**
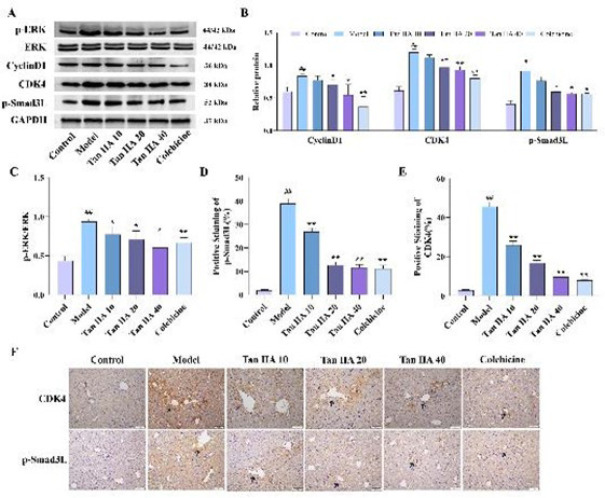
Expression of ERK/cyclin D1/p-Smad3L axis-related proteins *in vivo*

The related protein expression in mice liver tissues was examined using western blotting and immunohistochemistry experiments to gain insight into the relationship between Tan IIA inhibition of LF and ERK/cyclin D1/p-Smad3L axis. Compared to the control group, the phosphorylation of ERK was significantly higher in the model group (*P*<0.01) and significantly lower after drug application (*P*<0.05, Figure 3 A and C). Moreover, the expression levels of cyclin D1, CDK4, and p-Smad3L were significantly increased after CCl_4_ treatment (*P*<0.01). Tan IIA effectively reduced the expression of these proteins; the differences are statistically significant except at low doses (*P*<0.05, Figure 3 A and B). We also performed immunohistochemical analysis to observe the specific expression patterns of p-Smad3L and CDK4 in liver tissues (Figure 3 D-F). The positively stained areas of p-Smad3L and CDK4 showed a clear dose-dependent decrease with increasing doses of Tan II A (*P*<0.05).

### Effects of Tan IIA on the activation of HSCs and the formation of the ECM

**Figure 4:**
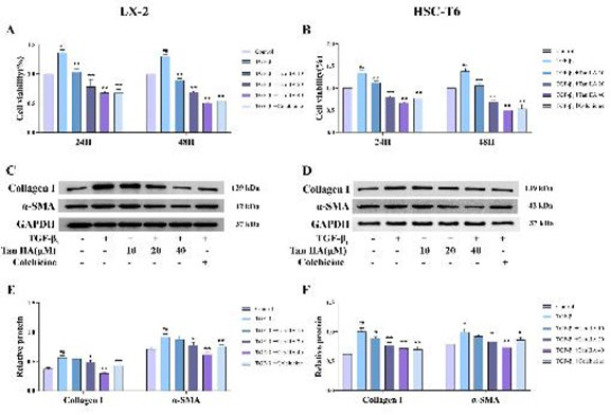
Effects of Tan IIA on HSCs activation and ECM formation

Cell viability was increased significantly after TGF-β_1 _stimulation (*P*<0.01); it was decreased markedly in Tan IIA groups (*P*<0.01). The inhibitory effect of Tan IIA was more pronounced at 24 hr (Figure 4 A and B). Next, we chose 24 hr as the treating time for the following experiments. The levels of α-SMA and collagen I were increased by TGF-β_1_ stimulating (*P*<0.01) and decreased by Tan IIA, especially for the high-dose group (*P*<0.01), in LX-2 (Figure 4 C and E) and HSC-T6 cells (Figure 4 D and F). 

### Effects of Tan IIA on colony formation and cell cycle of HSCs

**Figure 5:**
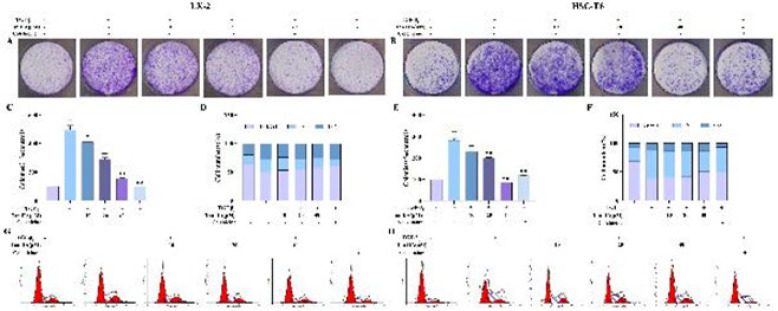
Influence of Tan II A on colony formation and cell cycle in LX-2 and HSC-T6 cells

The cells exhibited a strong clone formation ability in the cell clone formation experiment. The number of clones was significantly higher after TGF-β_1 _stimulation (*P* <0.01) and decreased significantly with different concentrations of Tan IIA (*P*<0.05, Figure 5 A, B, C, and E). In the cell cycle analysis, the cells in the G1 phase were reduced by TGF-β_1 _stimulation, while the G1 phase cell proportions were concentration-dependent and increased by Tan IIA treatment (Figure 5 D, F, G, and H). 

### Effects of Tan IIA on the protein expression of ERK/cyclin D1/p-Smad3L axis in TGF-β1-stimulated HSCs

**Figure 6:**
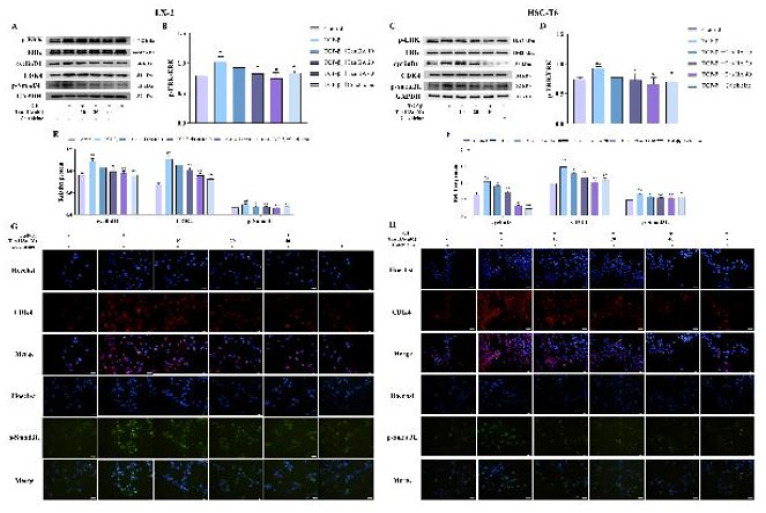
Effects of Tan IIA on ERK/cyclin D1/p-Smad3L axis protein levels in TGF-β1-treated HSCs

The expression levels of the p-ERK, cyclin D1, CDK4, and p-Smad3L protein were significantly increased in the TGF-β_1 _group (*P*<0.05). The levels of these proteins were effectively decreased by different concentrations of Tan IIA (*P*<0.05, Figure 6 A-F). The immunofluorescence results showed that the protein contents of CDK4 and p-Smad3L were significantly increased in the TGF-β_1_ group compared with the control group. In contrast, the expression of these two proteins was significantly reduced in the presence of Tan IIA, consistent with the western blotting results (Figure 6 G and H).

## Discussion

This study demonstrates that Tan IIA inhibits TGF-β_1_-mediated HSCs activation and proliferation, which depends on the ERK signaling pathway. Tan IIA inhibited the phosphorylation of the Smad3 linker region by affecting the binding of cyclin D1 and CDK4 through the ERK signaling pathway. In summary, we propose that Tan IIA may inhibit the proliferation of activated HSCs through the ERK/cyclin D1/p-Smad3L signaling axis, thereby reducing LF.

LF is a key stage in the development of chronic liver disease. If left untreated, it may progress to cirrhosis or even liver cancer, ultimately resulting in a high mortality risk (22). Therefore, finding effective drugs to treat LF and prevent its progression is crucial. Due to its complex pathogenesis, western medicine lacks effective LF therapies (23). Traditional Chinese medicine offers the advantages of diverse components, multiple targets, and minimal side effects. It has made good progress in the research of anti-LF (24). Tan IIA is an active ingredient extracted from the traditional Chinese medicine *S. miltiorrhiza*. It has anti-inflammatory, antioxidant, and anti-apoptotic effects and is widely used to treat cardiovascular, nervous, and intestinal diseases (25-27). Recent studies have shown that Tan IIA also has anti-LF effects (28, 29), but its precise mechanism of action remains unclear.

The development of LF is often linked to the activation of HSCs. HSC activation causes an imbalance between ECM synthesis and degradation(30, 31), leads to excessive collagen accumulation (primarily collagen I), and ultimately contributes to LF development (32). Therefore, the activation and transformation of quiescent HSCs into myofibroblasts is a key factor in LF development. During chronic liver injury, inflammatory factors or cytokines stimulate massive proliferation of HSCs, which exhibit high levels of α-SMA expression (33). High α-SMA expression is a typical feature of myofibroblasts and is the most reliable marker of HSC activation (34). Inhibiting HSCs activation and proliferation is a key focus in LF prevention and treatment. This study shows that CCl_4_ increases α-SMA expression to promote HSC activation and proliferation. Tan IIA significantly reduced the expression of α-SMA and col1α1 and alleviated the risk of LF in mice. *In vitro*, Tan IIA could inhibit the abnormal proliferation of activated HSCs due to TGF-β_1_ stimulation, and their number was significantly reduced. These results suggest that Tan IIA may exert an anti-hepatic fibrosis effect by inhibiting the activation and proliferation of HSCs and reducing ECM deposition.

TGF-β refers to a group of important profibrotic cytokines, which includes TGF-β_1_, TGF-β_2_, and TGF-β_3_. TGF-β_1_ is a key cytokine that activates HSCs to convert into myofibroblasts. It exerts its effects by targeting both classical Smad and non-classical Smad pathways, including JNK, ERK, p38, and PI3K signal pathways (35-37). As a key transcription factor of the TGF-β signaling pathway, Smad3 plays an important role in the pathophysiological process of fibrotic diseases. Inhibiting TGF-β/Smad3 signaling has emerged as a potential target for preventing and treating LF. When stimulated by TGF-β_1_, various signaling pathways phosphorylate Smad3 in distinct manners, leading to the formation of isoforms with either C-terminal or linker phosphorylation (38, 39). Phosphorylation of Smad3 in the linker region produces p-Smad3L, which generates a mitotic signal. This signal promotes the proliferation of activated HSCs, leading to fibrosis. Multiple kinases, primarily from the MAPK family, phosphorylate the link region of the Smad3 protein in various diseases. The ERK signaling pathway, a member of the MAPK signaling family, is abnormally activated during LF, causing dysregulation of some downstream transcription factors and promoting the proliferation and activation of HSCs (40). Furthermore, ERK-dependent p-Smad3L enhances the synthesis of Col-I under TGF-β stimulation (41). Our *in vivo* and *in vitro* results show that both phosphorylation of ERK1/2 and p-ERK/ERK ratio were increased. The ERK signaling pathway was activated, which resulted in the activation of p-Smad3L. Notably, our study reveals that Tan IIA suppresses the activation of these kinases, thereby preserving normal p-Smad3L value. These results further indicate that Tan IIA intervention in the fibrosis process may prevent the activation and proliferation of HSCs by blocking the non-classical TGF-β profibrotic signal transduction pathway.

Cell growth and proliferation are closely linked to cell cycle regulation. Cyclin D1 is specifically expressed during the G1 phase of the cell cycle, regulating the transition of cells from the G0 phase to the G1 phase. It is crucial for the initiation of the cell proliferation cycle. Continuous ERK phosphorylation activates the cyclin D1 expression, facilitates its complex formation with CDK4/6, and regulates the G1/S transition in the cell cycle process (42), promoting cells to enter into the S phase, potentially leading to excessive cell proliferation (43, 44). Studies indicated that Smad3 is the primary substrate for the G1 phase cell cycle-dependent kinases CDK4 and CDK2; CDK4 can inhibit its transcriptional activity and anti-proliferative function by phosphorylating the linker region of Smad3 (9, 45). Upon TGF-β stimulation, Smad3 can inhibit the cell cycle progress in the G1 phase. In addition, expression of mutant Smad3 (mutation at the linker region phosphorylation sites) in these cells inhibited proliferation more significantly than expression of wild-type Smad3 (46). In this study, Tan IIA prevented certain HSCs from staying in the G1 phase and inhibited the proliferation of LX-2 and HSC-T6. Does this inhibitory effect of Tan IIA affect the proliferation of HSCs function by associating ERK, cyclin D1, and p-Smad3L? The results show that the expression of p-Smad3L in the CCl_4_-induced mouse LF model and TGF-β_1_-activated HSCs was consistent with the cyclin D1 and CDK4 trend, and Tan IIA could significantly reduce their expression levels. The above results preliminarily suggest that Tan IIA may regulate CDK4 phosphorylation through the ERK signaling pathway and then affect the phosphorylation linker region of the Smad3 to exert an anti-proliferative effect. However, its impact on the specific downstream targets of p-Smad3L needs to be further investigated.

**Table 1:** Influences of Tan IIA on liver function in CCl_4_ treated mice

Group	Liver index/%	ALT/U·L^-1^	AST/U·L^-1^	LDH/U·L^-1^	γ-GT/U·L^-1^
Control	5.31 ± 0.099	12.512 ± 0.231	10.176 ± 4.377	1025.773 ± 234.454	13.723 ± 1.070
Model	5.81 ± 0.279^##^	337.270 ± 8.227^##^	188.303 ± 11.245^##^	4040.501 ± 123.700^##^	39.294 ± 4.132^##^
Tan ⅡA 10	5.69 ± 0.432	282.793 ± 8.990	165.275 ± 24.102	3672.680 ± 111.445	29.705 ± 3.164
Tan ⅡA 20	5.10 ± 0.190	269.981 ± 19.972	160.131 ± 13.020	3512.518 ± 114.398	21.523 ± 5.890
Tan ⅡA 40	5.01 ± 0.177	223.362 ± 38.661	154.262 ± 9.094	3143.041 ± 65.216	15.403 ± 3.462
Colchicine	4.93 ± 0.068	239.350 ± 29.621	158.594 ± 8.478	3518.041 ± 120.876	26.404 ± 4.410

Data are presented as mean ± SD; n = 8. ^#^*P*<0.05, ^##^*P*<0.01* vs* Control. *P<0.05, **P<0.01* vs* Model.

ALT: Alanine transaminase; AST: Aspartate transaminase; LDH: Lactate dehydrogenase; γ-GT: γ-glutamyl transpeptidase

(A) Liver morphology in different groups of representative C57BL/6J mice. (B) H&E staining of liver tissue sections from C57BL/6J mice. Black arrows indicate inflammatory cell infiltration and collagen deposition. (C) Masson trichrome staining of liver tissue sections from C57BL/6J mice. Black arrows indicate collagen depositions. (D) Sirius Red staining of liver tissue sections from C57BL/6J mice. Black arrows indicate collagen depositions. All these pathology images are shown, with the original magnification of ×200 and the scale bars representing 50 μm. (E) The relative stained area for Masson’s trichrome staining. Data are presented as mean ± SD; n = 8. (F) The relative stained area for Sirius Red. Data are presented as mean ± SD; n = 8. ^#^*P*<0.05, ^##^*P*<0.01 *vs* Control. *P<0.05, **P<0.01 *vs* Model.

(A) The total liver lysates were subjected to the western blot analysis of α-SMA and collagen I. GAPDH expression was measured as a loading control. (B) Quantification of α-SMA and collagen I protein content in liver tissue. Data are presented as mean ± SD; n = 3. (C) Immunohistochemistry staining of α-SMA and collagen I. α-SMA and collagen I proteins are indicated by black arrows, with the original magnification of ×200. (D) Quantitative analysis of the positive area of α-SMA and collagen I protein through immunohistochemical detection. The proportion of positively stained areas in different groups is calculated. Data are presented as mean ± SD; n = 3.

^#^ *P*<0.05,^ ##^*P*<0.01 *vs* Control. *P<0.05, **P<0.01 *vs* Model.

α-SMA: α-Smooth muscle actin; GAPDH: Glyceraldehyde 3-phosphate dehydrogenase

(A) (B) (C) Tan IIA significantly altered the expression of proteins such as p-ERK, cyclin D1, CDK4, and p-Smad3L, as evidenced by western blot analysis. GAPDH expression was measured as a loading control. (D) and (E) Quantitative CDK4 and p-Smad3L expression analysis was conducted through immunohistochemical analysis. The proportion of positively stained areas in different groups was calculated. Data are presented as mean ± SD; n = 3. (F) Immunohistochemistry staining of CDK4 and p-Smad3L in liver tissue. CDK4 and p-Smad3L proteins are indicated by black arrows, with the original magnification of ×200.

^#^ *P*<0.05, ^##^*P*<0.01 *vs *Control. *P<0.05, **P<0.01 *vs* Model. ERK: Extracellular signal-regulated kinase; p-ERK: Phospho-extracellular signal-regulated kinase; CDK4: Cyclin-dependent kinases4

(A) (B) LX-2 and HSC-T6 cells were cultured in varying doses of Tan IIA (10, 20, and 40 µM) and TGF-β_1 _(1.5 ng/ml) for 24 and 48 hr. Data are presented as mean ± SD; n = 3. (C) (D) (E) (F) LX-2 and HSC-T6 cells were treated with varying doses of Tan IIA (10, 20, and 40 µM) and TGF-β_1_ (1.5 ng/ml or not) for 24 hr. Representations of western blotting and relative protein expression of α-SMA and collagen I. GAPDH expression was measured as a loading control. Data are presented as mean ± SD; n = 3. ^#^*P*<0.05, ^##^*P*<0.01 *vs* Control. *P<0.05, **P<0.01 *vs* TGF-β1.

(A) (B) (C) (E) Influence of Tan II A and TGF-β1 on the clonogenic ability of LX-2 and HSC-T6 cells, as determined by a colony formation assay. The number of clonal clusters greater than or equal to 50 cells is calculated. Data are presented as mean ± SD; n = 3. (D) (F) Quantification of the cell cycle distribution of LX-2 and HSC-T6 cells. (G) (H) Alterations in cell cycle distribution induced by treatment with Tan II A or TGF-β1, assessed through flow cytometry. ^#^*P*<0.05, ^##^*P*<0.01 *vs* Control. *P<0.05, **P<0.01 *vs* TGF-β1.

(A), (B), (C) (D), (E), and (F) Representations of western blotting and relative protein expression of ERK, p-ERK, cyclin D1, CDK4, and p-Smad3L in LX-2 or HSC-T6 cells with TGF-β1 or Tan IIA treatment, GAPDH expression was measured as a loading control. Data are presented as mean ± SD; n = 3. (G) and (H) Immunofluorescence analysis of CDK4 (red) and p-Smad3L (blue) expression. The nuclei are visualized in blue. ^#^*P*<0.05, ^##^*P*<0.01* vs* Control. *P<0.05, **P<0.01 *vs* TGF-β1.

## Conclusion

In summary, this study reveals for the first time that Tan IIA may improve LF by inhibiting the ERK/cyclin D1/p-Smad3L signaling axis, which blocks activated HSCs in the G1 phase and suppresses their proliferation. This may provide new theoretical evidence for the application of Tan IIA in the treatment of LF.

## Declaration

We have not used any AI tools or technologies to prepare this manuscript.
